# Myocardial Integrated Backscatter in Obese Adolescents: Associations with Measures of Adiposity and Left Ventricular Deformation

**DOI:** 10.1371/journal.pone.0141149

**Published:** 2015-10-22

**Authors:** Lijian Xie, Elim Man, Pik-to Cheung, Yiu-fai Cheung

**Affiliations:** 1 Shanghai Children’s Hospital, Shanghai Jiaotong University, Shanghai, China; 2 Department of Paediatrics and Adolescent Medicine, Queen Mary Hospital, The University of Hong Kong, Hong Kong, China; Temple University, UNITED STATES

## Abstract

**Background:**

Myocardial fibrosis has been proposed to play an important pathogenetic role in left ventricular (LV) dysfunction in obesity. This study tested the hypothesis that calibrated integrated backscatter (cIB) as a marker of myocardial fibrosis is altered in obese adolescents and explored its associations with adiposity, LV myocardial deformation, and metabolic parameters.

**Methods/Principal Findings:**

Fifty-two obese adolescents and 38 non-obese controls were studied with conventional and speckle tracking echocardiography. The average cIB of ventricular septum and LV posterior wall was measured. In obese subjects, insulin resistance as estimated by homeostasis model assessment (HOMA-IR) and glucose tolerance were determined. Compared with controls, obese subjects had significantly greater cIB of ventricular septum (-16.8±7.8 dB vs -23.2±7.8 dB, p<0.001), LV posterior wall (-20.5±5.6 dBvs -25.0±5.1 dB, p<0.001) and their average (-18.7±5.7 dB vs -24.1±5.0 dB, p<0.001). For myocardial deformation, obese subjects had significantly reduced LV longitudinal systolic strain rate (SR) (p = 0.045) and early diastolic SR (p = 0.015), and LV circumferential systolic strain (p = 0.008), but greater LV longitudinal late diastolic SR (p<0.001), and radial early (p = 0.037) and late (p = 0.002) diastolic SR than controls. For the entire cohort, myocardial cIB correlated positively with body mass index (r = 0.45, p<0.001) and waist circumference (r = 0.45, p<0.001), but negatively with LV circumferential systolic strain (r = -0.23, p = 0.03) and systolic SR (r = -0.25, p = 0.016). Among obese subjects, cIB tended to correlate with HOMA-IR (r = 0.26, p = 0.07).

**Conclusion:**

Obese adolescents already exhibit evidence of increased myocardial fibrosis, which is associated with measures of adiposity and impaired LV circumferential myocardial deformation.

## Introduction

Childhood obesity has become a global epidemic involving not only developed but also developing countries.[1] Among the various co-morbidities, left ventricular (LV) dysfunction remains to be the most significant cause of morbidity and mortality.[2, 3] Importantly, myocardial fibrosis has been proposed to play an important pathogenetic role in ventricular dysfunction in obesity.[4–6] Experimental studies have provided evidence of dysregulation of the myocardial fibrotic process in obesity and metabolic dysfunction, which probably involves the activation of renin-angiotensin-aldosterone system and induction of oxidative stress.[7–9]

Whether increased myocardial fibrosis occurs in obese children is, however, unknown. Limited data suggest that this may be the case in obese adults. Myocardial biopsy to quantify the amount of fibrous tissue is invasive and impractical. On the other hand, noninvasive assessment of myocardial fibrosis by echocardiographically-derived calibrated integrated backscatter (cIB) has been used in both adults [10, 11] and children [12] to provide an estimate of myocardial fibrosis.

Alteration of myocardial composition and architecture may affect myocardial mechanics. Conventional echocardiographic assessment of LV function in obese subjects has nonetheless relied on measuring LV ejection fraction, which often yielded normal results.[13] On the other hand, direct interrogation of myocardial deformation compared with simple quantification of ventricular volume changes has been shown to be more sensitive in the early detection of subtle ventricular dysfunction.[14] In particular, two-dimensional strain imaging by speckle tracking echocardiography (STE) is increasingly used to evaluate ventricular performance in children with congenital [15,16] and acquired [17] heart diseases.

In the present study, we aimed to test the hypothesis that cIB is altered in obese adolescents compared with non-obese subjects. Furthermore, we explored its associations with measures of adiposity, indices of LV myocardial deformation, and, in obese adolescents, metabolic parameters including homeostasis model assessment of insulin resistance (HOMA-IR) and glucose tolerance.

## Methods

### Subjects

A total of 52 obese adolescents and 38 non-obese healthy adolescents aged 14 to 20 years were studied. Obese subjects were recruited consecutively from the paediatric clinic, while non-obese controls are friends of hospital staff and volunteers recruited from the community. The obese phenotype is defined by body mass index (BMI) at or above 95 percentile for children and adolescents of the same age and sex. Exclusion criteria included documented congenital or acquired heart diseases and current use of cardiovascular medications. Weight and height were measured and BMI was calculated accordingly. Waist circumference was measured at the midpoint between the lowest part of costal margin and highest point of the iliac crest in the mid-axillary line. Blood pressure of the right arm was measured three times with an automated oscillometric device (Dinamap, Critikon Inc, Tampa, USA) after the subjects had taken a rest for at least 15 minutes, and the average was used for analyses.

### Ethics statement

This study was approved by the Institutional Review Board of The University of Hong Kong/ Hospital Authority West Cluster, Hong Kong and informed written consent was obtained from all subjects and parents on behalf of minors.

### Conventional and tissue Doppler echocardiographic assessment

Echocardiographic examination was performed using Vivid 7 ultrasound machine (GE Medical System, Horten, Norway). Acquired data were stored digitally and analyzed offline using the EchoPAC software (GE Medical System, Horten, Norway). Average values of echocardiographic indices based on readings from three cardiac cycles were used for statistical analyses.

M-mode echocardiography was performed from the standard parasternal short-axis view for measurement of the following indices: LV end-systolic and end-diastolic dimensions, shortening fraction, septal and LV posterior wall thickness, and LV mass.

From the four-chamber view, pulsed-wave Doppler examination was performed to determine transmitral peak early (E) and late (A) diastolic velocities, E wave deceleration time, and E/A ratio. Colour tissue Doppler imaging of the LV lateral wall was performed with frame rates >100 Hz. With the sample volume positioned at the LV lateral wall-mitral annular junction, the mitral annular peak myocardial velocities at systole (s), early diastole (e), and late diastole (a) were measured and the mitral E/e ratio was also calculated. The relatively load-independent index of ventricular systolic function, myocardial acceleration during isovolumic contraction (IVA), was also measured as reported.[18]

### Speckle tracking echocardiography

Left ventricular myocardial deformation was assessed also using EchoPAC software (General Medical System, Horten, Norway). From the four-chamber view, LV global longitudinal systolic strain and systolic and diastolic strain rate (SR) were measured. Based on the mid-ventricular parasternal short-axis plane, LV global systolic circumferential and radial strain and systolic and diastolic SR were measured. Our group has previously reported on high intra- and inter-observer reproducibility of STE in measuring LV myocardial deformation.[19]

### Calibrated integrated backscatter

Integrated backscatter of the ventricular septum and posterior LV wall at the mid-ventricular level was determined at end-diastole from the parasternal short-axis view. The sample volume was tracked manually to maintain the same region throughout the heart cycle. Myocardial cIB was calculated as the difference between integrated backscatter at the two sites and that of the pericardium. The average cIB at the two sites was derived for correlation analysis. The intra- and interobserver variability for CIB measurement was 4.9% and 6.5%, respectively.

### Assessment of glucose tolerance and insulin resistance

Oral glucose tolerance test was performed in the obese group in the morning after overnight fasting after echocardiographic assessment. Normal and impaired glucose tolerance and diabetes mellitus were defined according to standard guidelines.[20] The HOMA-IR was calculated as fasting plasma glucose (in mmol/l) x fasting serum insulin (in mU/l) / 22.5.[21]

### Statistical Analysis

Data are presented as mean±SD unless otherwise stated. Absolute values of strain and strain rates are presented to facilitate interpretation and analyses. Demographic and echocardiographic parameters between obese and non-obese cohorts were compared using unpaired Student's t test. The LV mass was indexed by both body surface area and height^1.7^, the latter being reported to be more sensitive for identification of obesity-related LV hypertrophy.[22] Pearson correlation analysis was used to determine associations between myocardial cIB and measures of body adiposity, indices myocardial deformation, and metabolic parameters. Adjustments of correlations for systemic blood pressure were made by linear regression analysis. A p value <0.05 is considered statistically significant. All statistical analyses were performed using SPSS version 16.0 (SPSS Inc, Chicago, Illinois, USA).

## Results

### Subjects

The 52 (25 males) obese adolescents were studied at 17.8±1.7 years, while the 38 (17 males) non-obese controls were studied at 18.1±2.1 years (p = 0.39). Table 1 summarizes their clinical characteristics. The BMI (p<0.001) and waist circumference (p<0.001) were expectedly greater in the obese cohort. Systolic (p<0.001) and diastolic (p<0.001) blood pressures were significantly higher in obese subjects compared with non-obese ones. Of the 52 obese subjects, 38 had normal glucose tolerance, 9 had type 2 diabetes mellitus, and 5 had impaired glucose tolerance.

**Table 1:** Clinical characteristics of obese and non-obese subjects.

	Obese subjects (n = 52)	Non-obese subjects (n = 38)	p
**Age (year)**	17.8±1.7	18.1±2.1	0.39
**Male / Female**	25/27	17/21	0.76
**Height (cm)**	167.5±8.8	164.3±7.3	0.21
**Weight (kg)**	87.5±13.2	53.2± 8.5	<0.001*
**BMI (kg/m** ^**2**^ **)**	31.5±3.7	19.6±2.1	<0.001*
**Waist circumference (cm)**	97.1±8.6	65.6±6.1	<0.001*
**SBP (mmHg)**	125±13	108±10	<0.001*
**DBP (mmHg)**	67±6	62±5	<0.001*
**fasting glucose (mmol/l)**	4.9±1.2		
**fasting insulin (mU/l)**	24.0±15.4		
**HOMA-IR**	5.0±3.2		

Abbreviations: BMI, body mass index, DBP, diastolic blood pressure, HOMA-IR, homeostasis model assessment of insulin resistance, SBP, systolic blood pressure

*statistically significant

### Conventional and Doppler echocardiographic parameters

Table 2 summarizes the echocardiographic parameters of the two groups. Compared with controls, obese subjects had significantly greater septal (p<0.001) and LV posterior wall (p<0.001) thickness at diastole and LV mass (p<0.001) even with adjustment of the latter for body surface area (p<0.001). The LV end-diastolic (p<0.001) and systolic (p = 0.006) dimensions were significantly greater in patients than controls, while their shortening fraction (p = 0.26) were similar.

**Table 2:** Comparison of echocardiographic indices between obese and non-obese adolescents.

		Obese subjects (n = 52)	Non-obese subjects (n = 38)	p
***M-mode***	**LV EDD (mm)**	48.6±4.7	43.9±4.3	<0.001*
	**LV ESD (mm)**	31.5±4.6	29.0±3.6	0.006*
	**IVSd (mm)**	7.8±1.4	6.0±0.9	<0.001*
	**LV PWd (mm)**	7.3±1.2	5.9±0.9	<0.001*
	**LV shortening fraction (%)**	35.2±4.9	34.0±5.2	0.26
	**LV mass (g)**	138.4±41.1	81.6±27.3	<0.001*
	**BSA-indexed LV mass (g/m** ^**2**^ **)**	68.5±16.8	51.9±15.3	<0.001*
	**LV mass/height** ^**1.7**^ **(g/m)**	57.9±15.1	34.8±10.8	<0.001*
***Transmitral velocities***	**E (cm/s)**	92.7±14.8	88.7±13.9	0.20
	**A (cm/s)**	46.9±9.1	41.6±8.9	0.008*
	**E/A ratio**	2.1±0.5	2.2±0.5	0.11
	**E deceleration time(ms)**	117.3±19.3	115.8±20.0	0.73
***Mitral annular tissue velocities***	**s (cm/s)**	6.6±1.3	7.27±1.36	0.016*
	**e (cm/s)**	10.4±2.6	10.75±2.01	0.45
	**a (cm/s)**	5.4±1.3	4.71±1.54	0.026*
	**e/a ratio**	2.0±0.8	2.4±0.7	0.013*
	**E/e Ratio**	9.5±2.7	8.5±1.7	0.034*
	**IVA (cm/s** ^**2**^ **)**	1.01±0.49	0.99±0.48	0.83

Abbreviations: A, transmitral late diastolic velocity, a, mitral annular late diastolic tissue velocity, BSA, body surface area, E, transmitral early diastolic velocity, e, mitral annular early diastolic tissue velocity, EDD, end-diastolic dimension, ESD, end-systolic dimension, IVSd, interventricular septal thickness at diastole, IVA, myocardial acceleration during isovolumic contraction, LV, left ventricular, PWd, posterior wall thickness at diastole

*statistically significant

For Doppler parameters, obese subjects had significantly greater transmitral A velocity (p = 0.008), mitral annular a velocity (p = 0.026), and E/e ratio (p = 0.034), but lower mitral annular s velocity (p = 0.016) and e/a ratio (p = 0.013).

### Myocardial cIB

**Fig 1:**
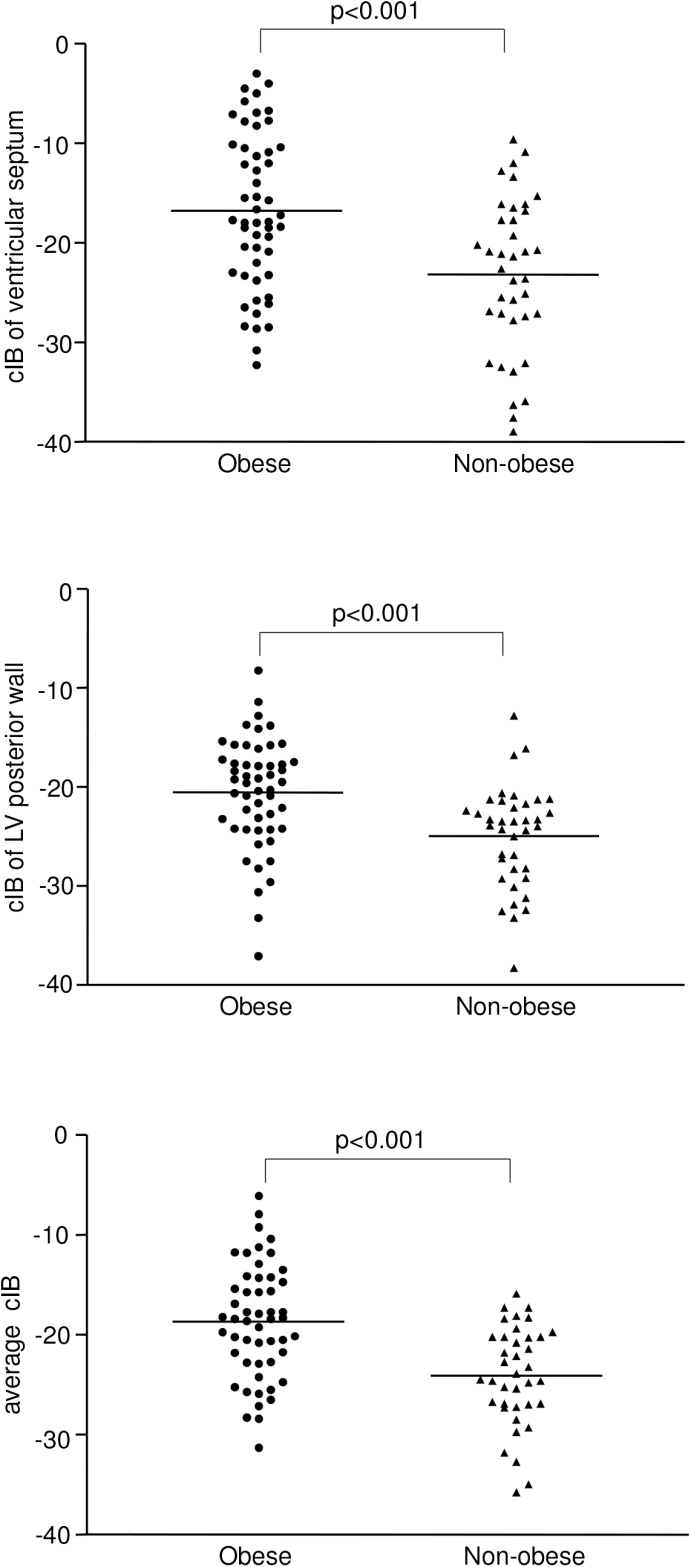
Scatter plots showing calibrated integrated backscatter (cIB) of ventricular septum and left ventricular (LV) posterior wall and the average of the two sites.

Myocardial cIB of the ventricular septum (-16.8±7.8 dB vs -23.2±7.8 dB, p<0.001), LV posterior wall (-20.5±5.6 dB vs -25.0±5.1 dB, p<0.001) and average cIB of the two sites (-18.7±5.7 dB vs -24.1±5.0 dB, p<0.001) were significantly greater in obese compared with non-obese subjects (Fig 1). Even with exclusion of the 9 obese subjects with diabetes mellitus, the remaining 43 obese subjects had similarly greater average cIB compared with controls (-18.87±5.43 dB vs -24.08±4.97 dB, p<0.001).

### Myocardial deformation

**Fig 2:**
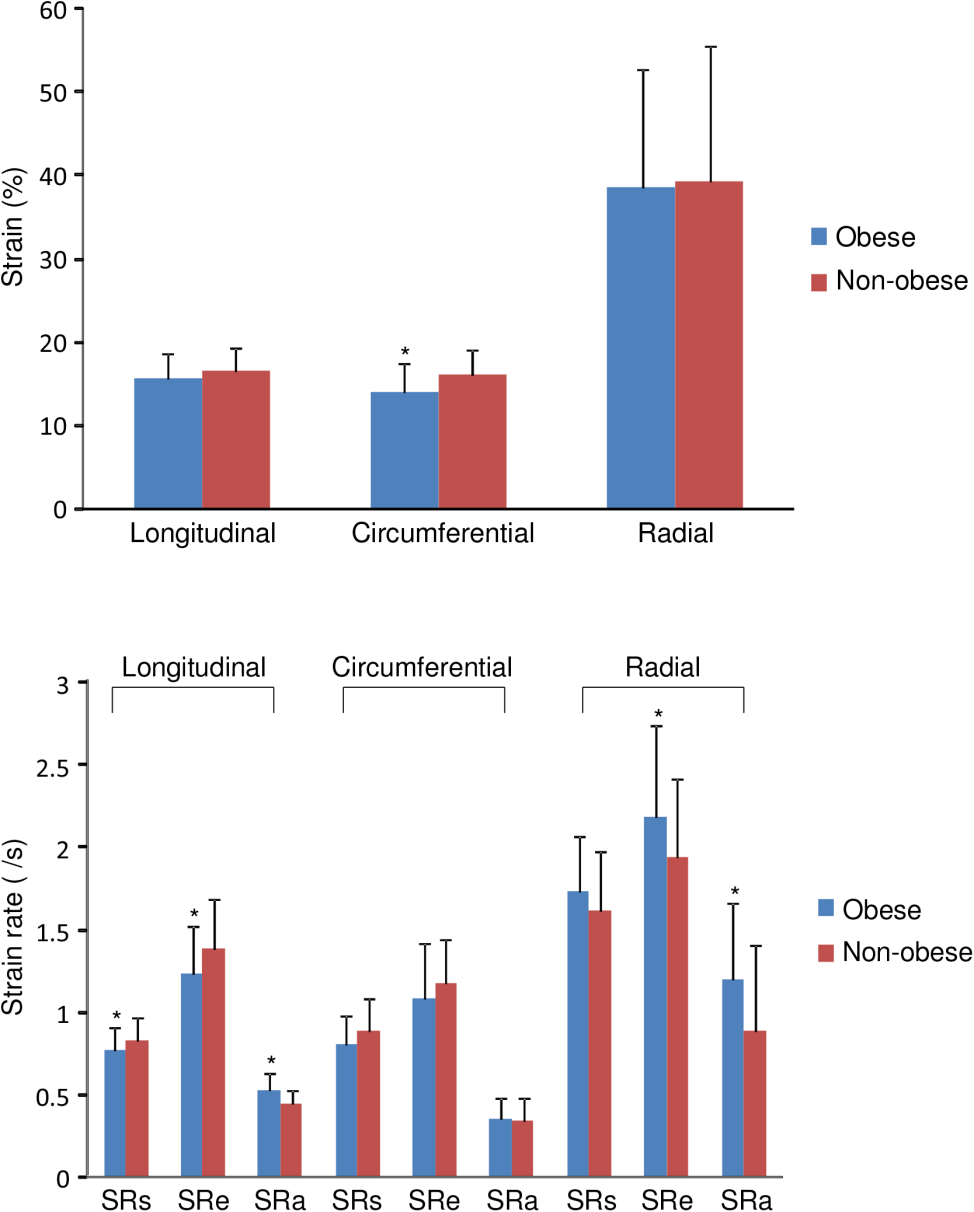
Comparison of myocardial systolic strain and systolic (SRs), early diastolic (SRe), and late diastolic (SRa) strain rate in three dimensions between obese and non-obese subjects (*p<0.05 vs controls).

Fig 2 shows the strain and strain rate of the two groups in three dimensions. Compared with controls, obesity subjects had significantly reduced LV longitudinal systolic SR (0.77±0.14 /s vs 0.83±0.14 /s, p = 0.045) and early diastolic SR (1.23±0.29 /s vs 1.38±0.30 /s, p = 0.015), and LV circumferential systolic strain (14.1±3.4% vs 16.0±3.1%, p = 0.008). On the other hand, obese subjects had significantly greater LV longitudinal late diastolic SR (0.52±0.11 /s vs 0.44±0.09 /s, p<0.001), and radial early (2.18±0.56 /s vs 1.94±0.48 /s, p = 0.037) and late (1.20±0.46 /s vs 0.88±0.53 /s. p = 0.002) diastolic SR (Fig 2).

When stratified by the presence (n = 14) or absence (n = 38) of glucose intolerance in obese subjects, no differences in myocardial deformation parameters were found (Table 3).

**Table 3:** Left ventricular deformation and fibrosis in obese subjects with normal and impaired glucose tolerance.

		Normal glucose tolerance (n = 38)	Abnormal glucose tolerance (n = 14)	p
***Longitudinal deformation***	**Strain (%)**	15.8±2.7	15.1±3.7	0.43
	**SRs (/s)**	0.78±0.13	0.75±0.18	0.48
	**SRe (/s)**	1.23±0.26	1.20±0.36	0.72
	**SRa (/s)**	0.52±0.10	0.53±0.13	0.77
***Circumferential deformation***	**Strain (%)**	14.1±3.5	13.9±3.3	0.84
	**SRs (/s)**	0.80±0.18	0.80±0.18	0.95
	**SRe (/s)**	1.07±0.34	1.11±0.38	0.76
	**SRa (/s)**	0.35±0.14	0.34±0.11	0.87
***Radial deformation***	**Strain (%)**	40.6±14.8	33.3±11.2	0.10
	**SRs (/s)**	1.75±0.37	1.66±0.27	0.40
	**SRe (/s)**	2.20±0.61	2.14±0.43	0.73
	**SRa (/s)**	1.19±0.46	1.23±0.47	0.80
***Myocardial Fibrosis***	**cIB**	-18.5±5.5	-19.3±6.4	0.67

Abbreviations: cIB, calibrated integrated backscatter, SRa, late diastolic strain rate, SRe, early diastolic strain rate, SRs, systolic strain rate

On the other hand, there were greater correlations between waist circumference and myocardial deformation parameters than those between BMI and the latter (Table 4). Even after adjustment for systolic and diastolic blood pressures, the waist circumference was found to correlate negatively with LV longitudinal systolic strain (p = 0.027), systolic SR (p = 0.02), early diastolic SR (p = 0.004) and circumference systolic strain (p = 0.007) and systolic SR (p = 0.05) and positively with radial late diastolic SR (p = 0.002).

**Table 4:** Correlations between measures of adiposity and left ventricular deformation adjusted for systolic and diastolic blood pressure.

		Body Mass Index		Waist Circumference	
		β	p	β	p
***Longitudinal deformation***	**Strain**	-0.29	0.17	-1.22	0.027*
	**SRs**	-7.05	0.09	-25.52	0.020*
	**SRe**	-4.76	0.017*	-15.07	0.004*
	**SRa**	10.55	0.05	26.50	0.07
***Circumferential deformation***	**Strain**	-0.50	0.006*	-1.28	0.007*
	**SRs**	-6.05	0.06	-16.26	0.05
	**SRe**	-2.64	0.17	-8.53	0.09
	**SRa**	0.46	0.92	-0.79	0.95
***Radial deformation***	**Strain**	-0.02	0.61	-0.03	0.80
	**SRs**	2.66	0.12	4.79	0.29
	**SRe**	1.13	0.32	2.99	0.31
	**SRa**	3.63	0.002*	9.30	0.002*

Abbreviations as in Table 2.

*statistically significant

### Correlates of cIB

**Fig 3:**
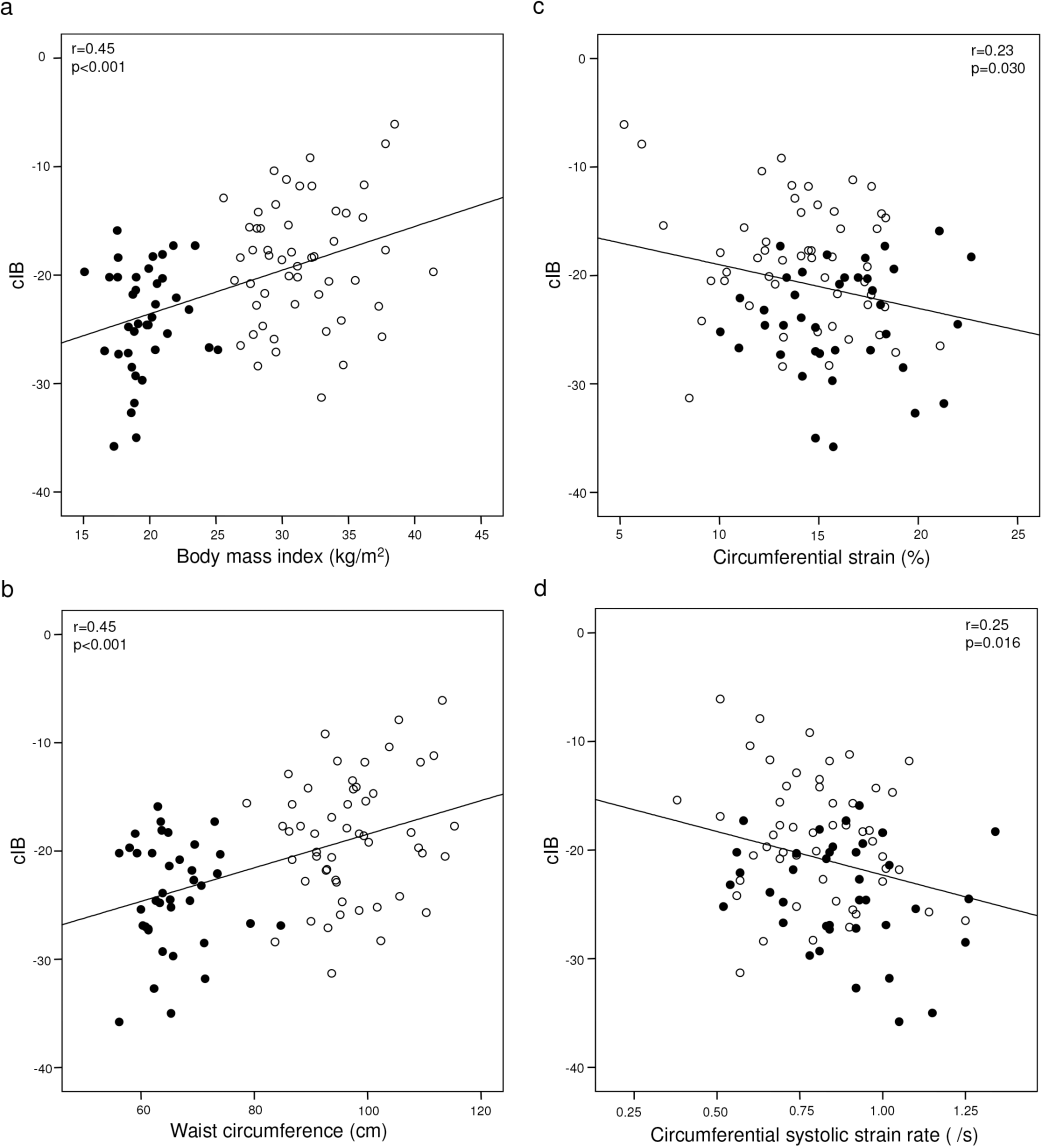
Scatter plots showing correlations between myocardial calibrated integrated backscatter and (a) body mass index, (b) waist circumference, (c) circumferential strain, and (d) circumferential systolic strain rate (empty circles represent obese subjects, solid circles represent non-obese controls).

For the entire cohort, the average myocardial cIB correlated positively with BMI (r = 0.45, p<0.001), waist circumference (r = 0.45, p<0.001) and negatively with LV circumferential systolic strain (r = -0.23, p = 0.030) and systolic SR (r = -0.25, p = 0.016) (Fig 3), but not with other deformation parameters. The correlations between cIB and BMI, waist circumference, and circumferential systolic SR remained significant even after exclusion of obese subjects with diabetes mellitus (all p<0.05). Among obese subjects, cIB also tended to correlate positively with HOMA-IR (r = 0.26, p = 0.07).

## Discussion

The present study demonstrates increased myocardial cIB suggestive of myocardial fibrosis and impaired LV myocardial deformation along the longitudinal and circumferential dimensions in obese adolescents. Additionally, myocardial cIB was found to associate positively with waist circumference and BMI and negatively with LV circumferential deformation.

In rodent models of obesity, cardiac remodeling with ventricular hypertrophy and increased interstitial fibrosis have been described.[5, 23, 24] Direct histological evidence of myocardial fibrosis in human obese subjects is, however, limited. Nevertheless, previous clinical studies have provided evidence of altered myocardial extracellular matrix turnover with possible predisposition to increased interstitial fibrosis in adults. In premenopausal obese woman, altered circulating profile of matrix metalloproteinases and their tissue inhibitors has been described,[25] although the absence of histological specimens forbids prediction of the net result on extracellular matrix turnover in this study. On the other hand, procollagen type III aminopeptide, a circulating marker of collagen synthesis, was found to be increased in obese adults and to correlate positively with the severity of insulin resistance.[6] Recently, a randomized controlled trial of aldosterone blockade in middle-aged obese adults showed reduction of circulating procollagen levels coupled with improved myocardial cIB and LV function, implicating a possible pathogenetic role of myocardial fibrosis.[26] Little, however, is known on the myocardial fibrotic process in obese children. To our knowledge, this is the first study to interrogate myocardial fibrosis and its association with measures of adiposity and myocardial deformation in obese children. The finding of greater myocardial cIB in obese adolescents suggests that increased fibrosis of the myocardium starts since childhood.

Changes in extracellular matrix composition may have functional implications on LV mechanics. Previous studies on LV systolic function in obese subjects by quantification of ejection fraction usually yielded normal results.[13] On the other hand, assessment of myocardial mechanics by strain imaging may provide better assessment of ventricular contractility.[14] The findings of impaired LV systolic deformation along the longitudinal and circumferential dimensions in the obese cohort agree with those reported previously.[27, 28] Our additional findings of negative correlations between myocardial cIB and circumferential systolic strain and SR probably represent functional translation of altered myocardial extracellular matrix. These findings corroborate the associations between cIB and basal septal strain and SR reported in adults with metabolic syndrome [11] and provide evidence of interplay between myocardial fibrosis and systolic dysfunction in obesity. Interstitial fibrosis has indeed been linked to impaired LV contractile performance in the setting of aortic regurgitation,[29] while perivascular fibrosis may reduce coronary flow reserve to cause contractile dysfunction.[30]

Fibrous tissue deposition may increase myocardial stiffness and lead to ventricular diastolic dysfunction. This is the first study to explore diastolic myocardial deformation using STE in obese children. While we did not find statistically significant correlations between cIB and indices of diastolic function, reduced LV longitudinal early diastolic SR and higher E/e ratio in our obese cohort may reflect respectively ventricular relaxation abnormality and greater LV filling pressure. The latter concurs with findings of previous studies in obese children.[27, 31] Furthermore, the greater late diastolic transmitral and mitral annular velocities and late diastolic longitudinal and radial SR implicate greater reliance on atrial filling in obese adolescents. Reduced atrial deformation reported in obese children [31] may, however, limit the degree of atrial compensation. The discrepant association between calibrated integrated backscatter with ventricular systolic and diastolic deformation may perhaps be related to the degree of myocardial fibrosis. Mild fibrosis may not significantly alter ventricular compliance and diastolic function, although subtle subclinical alteration of systolic deformation may already be detectable using the sensitive speckle tracking echocardiography.

Associations of measures of body adiposity, in particular waist circumference, with myocardial cIB and deformation indices even after adjustment of systemic blood pressure are of clinical importance. Body mass index and waist circumference, with the latter having a greater sensitivity, can predict clustering of traditional cardiovascular risk factors in children.[32, 33] Our findings suggest potential inclusion of subclinical LV dysfunction to this list of risk factors. While the mechanistic link between adiposity and myocardial fibrosis in our subjects is not entirely clear, experimental studies have shown that the adipokine leptin could induce cardiac fibrosis through activation of the mTOF pathway, increase in oxidative stress, and finally production of galectin-3 to stimulate collagen deposition.[9] The negative associations between adiposity and longitudinal and circumferential deformation found in this study corroborate the findings of Peterson et al, who reported on negative associations between BMI and systolic and early diastolic myocardial velocities in obese women.[34] Whether the observed ‘dose-response’ relationships between adiposity and subtle subclinical LV systolic and diastolic dysfunction is directly related to cardiac remodeling and alteration of myocardial extracellular matrix or indirectly through the effects of systemic hypertension require further clarification.

Within our relatively small obese paediatric cohort, the HOMA-IR tended to correlate with myocardial cIB. As aforementioned, in normotensive, non-diabetic obese adults, circulating procollagen type III aminopeptide has been shown to be associated with insulin resistance.[6] Recent cardiac magnetic resonance contrast-enhanced T1 mapping has also provided evidence that diffuse myocardial fibrosis in diabetic patients contributes to systolic and diastolic ventricular dysfunction.[35, 36]

Several limitations to this study warrants discussion. Firstly, the small number of patients with glucose intolerance and diabetes limits the statistical power to perform subgroup analysis. Further large scale studies of paediatric obese subjects are required to clarify the relationships between markers of myocardial fibrosis, insulin resistance, and glucose homeostasis. Secondly, the modality of T1 mapping, which is ideal for assessment of diffuse myocardial fibrosis, is not available to us. It is worth noting, however, that conditions other than fibrosis may also influence extracellular volume measurements by T1 mapping.[37] Thirdly, we have not determined circulating markers of collagen synthesis in the present study. The cardiac origin of these collagen synthesis biomarkers is difficult to ascertain. More importantly, their levels vary with age and growth,[38] which may confound the interpretation in paediatric studies. Allometric indexation using approximate powers has been shown to allow more accurate normalization of LV mass [22] and quantification of arterial load and ventricular-arterial coupling [39] in obese subjects. It would have been ideal to adopt a similar approach in this study. However, a large scale study to define a priori the normal relationships between parameters of myocardial deformation and body size is required before allometric indexation is possible. We have nonetheless compared the BMI indexed in an allometric manner between the two groups and showed significant differences. Finally, differences among ethnic groups may potential exist due to interactions of genetic, physiological, cultural, socioeconomic, and environmental factors in the pathogenesis of obesity.[40, 41] Whether the results of the present study apply to obese adolescents of other ethnicities require further clarification.

In conclusion, our findings suggest increased myocardial fibrosis in obese adolescents, which is related to measures of adiposity and may have implications on subclinical LV systolic and diastolic function. Whether weight reduction and anti-fibrotic strategies such as aldosterone antagonism may alter myocardial acoustic properties and improve myocardial deformation in young subjects are topics for further studies.
